# Field screening and identification of biochemical indices of pod borer (*Helicoverpa armigera*) resistance in chickpea mutants

**DOI:** 10.3389/fpls.2024.1335158

**Published:** 2024-05-10

**Authors:** Asima Noreen, Amjad Hameed, Tariq Mahmud Shah

**Affiliations:** Nuclear Institute for Agriculture and Biology College, Pakistan Institute of Engineering and Applied Sciences (NIAB-C, PIEAS), Faisalabad, Pakistan

**Keywords:** pod borer, enzymatic antioxidants, non-enzymatic antioxidants, *Cicer arietinum* L., trichome

## Abstract

Chickpea pod borer (CPB) (*Helicoverpa armigera*) is one of the major pests, causing significant yield losses. The objectives were to screen chickpea mutants for pod borer resistance/tolerance under field conditions and identification of biochemical markers of tolerance. Chickpea mutant CM216-A/15 had highest leaf (25 trichomes/mm^2^) and stem trichome density (17 trichomes/mm^2^) with least pod damage at Kallur Kot and highest pod weight per plant (22.8 ± 2.6g) at AZRI. Higher total phenolic contents (TPCs) and antioxidant capacity were detected in tolerant mutants, i.e., CM216-A/15 and CM664/15. TPC was positively associated with pod yield and had negative correlation with pod damage. Mutants CM216-A/15, CM664/15, and CM766/15 depicted the highest resilience to CPB, owing to higher hairiness, better antioxidant defense response, and lower levels of hydrolytic enzymes and sugars. Identified biochemical markers like TPC, total oxidant status, superoxide dismutase, and pigments can be used for screening of CPB-tolerant/resistant mutants.

## Introduction

Grain legumes are occasionally said as the poor man’s meat because of their essential nutritional role in the diets of lots of people in underprivileged countries. Beans are vital sources of phosphorus, calcium, iron, and proteins and, therefore, are an important portion of a vegetarian’s diet (Kumara Charyulu and Deb, 2014). Chickpea (*Cicer arietinum* L.) is preferred over other pulses in various locations due to its versatility. Chickpeas have higher content of protein (up to 40%). Furthermore, chickpea may provide health benefits that include lowering the risk of cardiac disorder, diabetes, and cancer (Merga and Haji, 2019). After peas and soybeans, chickpea is the third most significant pulse, accounting for roughly 15% of global pulses production (Iriti and Varoni, 2017). According to Pakistan Econmic survey 2021–2022, chickpea was grown on 867 thousand hectares in Pakistan, contributing to the production of 319 thousand tons, and contributes in the pulses share of 4.41% toward gross domestic product (Pakistan, 2022). It is primarily grown in the Thal areas of Punjab and Kyber Pakhtunkhwa provinces, where it is rainfed. The crop is cultivated on residual moisture after rice harvest in Sindh and Baluchistan. The Punjab province alone produces around 80% of the country’s chickpeas, with 90% of the country’s chickpeas being cultivated in rainfed circumstances (Hussain et al., 2015).

A large number of biotic/abiotic factors influence chickpea yield, just as they do for other crops. Diseases such as Ascochyta blight, Botrytis gray mold, Fusarium wilt, as well as root rot and stunt are among the biotic stresses, whereas major pests include *Helicoverpa armigera*, aphid, black cutworm, semilooper, bruchids, and leaf miner (Gurjar et al., 2011). The pod borer, *Helicoverpa armigera* (Hubner), is considered as the most destructive insect pest, causing 30%–40% damage to pods on average, which can climb to 80%–90% under favorable circumstances and can cause more than 75% of the yield losses in case of severe attack (Akbar et al., 2018). Three large pod borer outbreaks have occurred in the last decade, resulting in yield losses of 10%–80% due to pod destruction (Patil et al., 2017). The immediate lessening in crop production, monitoring cost and treating way of insect pests, result in monetary losses. Annually, chickpea losses in the semi-arid tropics have been estimated at about US$328 million (Patil et al., 2017). *Helicoverpa*-related losses in cotton, legumes, vegetables, grains, and other crops are more than US$2 billion every year, whereas more than US$1 billion is spent to combat these damaging pests annually (Mahmood et al., 2021).

Given the abovementioned, it is vital to handle the pest in a more environment-friendly manner. Sustainable pest management strategies like resistant varieties, many agronomic approaches, biological control actions, as well as integrated practices have all been explored. Various morphological traits have been used to develop pod borer–resistant cultivars of chickpea (Golla et al., 2018). Morphological factors such as length and density of pod trichome, thickness of wall, pod length, breadth as well as area, and total number of pods per plant have an impact on resistance of pod borer (Brar and Singh, 2017). In some crops, various types of trichomes, as well as their direction, length, and density have been linked to the reduced damage caused by insects (Sharma et al., 2009). The relationship between pod wall thickness and pod borer damage was shown to be negative, implying that chickpea mutants with thicker pod walls were less susceptible to pod borer damage. The resistance mechanism of chickpea against pod borer was also reported to be linked with the pod length and its area and breadth (Patil et al., 2017).

The dietary components of the host plant species, the effects of its morphological characteristics, and the secondary metabolites on herbivores are what lead to interactions between plants and herbivores (Golla et al., 2020). The host plant’s primary and secondary metabolites have an impact on the behavior, survival, and growth of the insects. Whereas the impact of primary metabolites or dietary variables depends upon the quantities of various constituents, the secondary metabolites reduce the digestibility of various plant tissues in the stomach of insect and change the larval stage of growth and development. The inadequate ratio of carbohydrates to proteins inhibits the development and growth of insects (Roeder and Behmer, 2014). It is well-known that chickpeas reduce the function of intestinal proteinase. As opposed to the cultivated chickpea, wild relatives of the chickpea have a wide variety of protease inhibitor isoforms. Phenols, one of the secondary metabolites, contribute significantly to the development of insect pest resistance by badly influencing larval stage of growth and development through mechanism of feeding inhibition and/or decreased metabolite production of larval stage. High phenol content is frequently cited as the cause of plant resistance to insect pest development stage. The negative effects of tannins on certain insects can range in severity from having no visible effects to stunted growth and development to eventual insect death. However, the concentration and chemical makeup of the tannins as well as the pH and antioxidant content in the insect gut determine the effects of tannins in the gut (Ballhorn et al., 2011). The host plant’s phenylpropanoid pathway is used to biosynthesize flavonoid chemicals, which have an impact on insect development, survival, and eating. Widely present in crop plants and assisting in herbivore tolerance are flavonoid chemicals such quercetin, chlorogenic acid, and rutin (Diaz Napal et al., 2010).

In order to identify the biochemical components contributing to resistance of host plant to *H. armigera* and use them as a criteria of selection to develop chickpea cultivars with constant and high resistance level, it is crucial to have a basic knowledge of the interactions among the biochemical characteristics of chickpea and growth and development stage of *H. armigera.* Developing insect-resistant cultivars gives a solid platform for the growth and development of integrated insect–pest management system. The reduction in insect numbers achieved by the adoption of highly resistant varieties of plants is consistent and collective, and it costs farmers essentially nothing more. As a result, the goals of breeding should be to find, describe, and use a genetic phenomena’s that imparts long-term pod borer resistance (Barmukh et al., 2021). If a good resistance source is obtainable and an effective and applied screening method that can offer a very good selection pressure exists, then developing better cultivars with highly resistance to pod borer is much simpler. Mutation breeding could be utilized to generate novel diversity for traits that have a favorable impact on pod borer resistance. Insect–pest resistance breeding yields of $300 for every $1 invested in research report (Brar and Singh, 2015). The antixenosis/antibiosis and avoidance processes are both involved in the breeding approach of chickpea to pod borer resistance. The avoidance technique can be used in conjunction with the antixenosis/antibiosis method (i.e., selection of chickpea mutants having capacity to set seed under very low-temperature managements or early-maturing chickpea mutants). Huge genetic difference for these phenological features has been identified, which the breeder can employ to reduce pod borer damage in chickpea (Sree Latha and Sharma, 2018).

The present study was conducted with objective to identify high yielding chickpea mutants with resistance/tolerance to pod borer. The other objective was to identify the metabolic and biochemical traits associated with pod borer tolerance in chickpea that can be used to devise a quick screening and selection protocol for resistant mutants.

## Materials and methods

### Experimental materials and sites

In this study, 30 chickpea lines including mutants (M_5_) and local standard varieties (CM-2008, NIAB CH2016) were screened on the occurrence, severity, and damage infestation of *Helicoverpa armigera* (chickpea pod borer CPb) under field conditions (Lateef, 1985). Experimental material was collected from the Chickpea group of Plant Breeding and Genetics Division, Nuclear Institute for Agriculture and Biology (NIAB), Faisalabad, Pakistan (**Table 1**). Experiment was conducted on four different sites in Pakistan (**Table 2**).

**Table 1:** Details about the origin and parentage of chickpea mutants and approved check varieties used in the study.

Sr. No.	Genotypes	Parent	Radiation Dose/Mutagen Dose	Generation
1.	CM8/15	09024	400 Gy	M_5_
2.	CM17/15	09024	400 Gy	M_5_
3.	CM26/15	09024	400 Gy	M_5_
4.	CM110/15	09024	500 Gy	M_5_
5.	CM113/15	09024	500 Gy	M_5_
6.	CM119/15	09024	500 Gy	M_5_
7.	CM126/15	09024	500 Gy	M_5_
8.	CM137/15	09024	500 Gy	M_5_
9.	CM173-A/15	09024	500 Gy	M_5_
10.	CM179/15	09024	500 Gy	M_5_
11.	CM208/15	09024	500 Gy	M_5_
12.	CM216-A/15	09024	500 Gy	M_5_
13.	CM269/15	09024	0.3% EMS	M_5_
14.	CM286/15	09024	0.3% EMS	M_5_
15.	NIAB CH2016	96052× Pb2000	–	Variety
16.	CM562/15	70005	200 Gy	M_5_
17.	CM583/15	70005	200 Gy	M_5_
18.	CM609/15	70005	200 Gy	M_5_
19.	CM641/15	70005	300 Gy	M_5_
20.	CM664/15	70005	0.1% EMS	M_5_
21.	CM698/15	70005	0.1% EMS	M_5_
22.	CM717/15	70005	0.1% EMS	M_5_
23.	CM752/15	70005	0.2% EMS	M_5_
24.	CM766/15	70005	0.2% EMS	M_5_
25.	CM785/15	70005	0.2% EMS	M_5_
26.	CM788/15	70005	0.2% EMS	M_5_
27.	CM797/15	70005	0.2% EMS	M_5_
28.	CM817/15	70005	0.2% EMS	M_5_
29.	CM850/15	70005	0.2% EMS	M_5_
30.	CM2008	Punjab-1	0.1% EMS	Variety

EMS, ethyl methanesulfonate.

**Table 2:** Details of experimental sites used for field trails.

Sr.	Site	Name	Location
1	Site 1	Nuclear Institute for Agriculture and Biology (NIAB), Faisalabad (Punjab), Pakistan	31.3989°N, 73.0331°E
2	Site 2	Gram Breeding Research Substation AARI, Kallur Kot, Bhakkar District, Punjab, Pakistan	32.1575°N, 71.2696°E
3	Site 3	Nuclear Institute for Agriculture (NIA), Tando Jam, Hyderabad District, Sindh, Pakistan	25.4216°N, 68.5415°E
4	Site 4	Arid Zone Research Institute (AZRI), Bhakkar District, Punjab, Pakistan	31.6344°N, 71.1202°E

### Field screening and morphological traits

Under field conditions, data were recorded for mean larval population of crop pod damage (CPD) (%), damage after harvest, and mean pod weight (g) per plant. Moreover, trichome density (hairiness) of stem and leaves was also recorded for each chickpea genotype and expressed in trichomes/mm^2^. The data were analyzed using appropriate statistical tools.

### Trichome density of chickpea leaves

Thirty chickpea mutant’s leaves were examined for trichome density using the methodology outlined by Jackai and Oghiakhe (1989) with some modifications (Golla et al., 2018). After being cut with scissors, the leaves were put in stoppered glass vials (10-mL capacity) containing acetic acid and alcohol (2:1) for 24 h to remove the chlorophyll. Under a stereomicroscope with 10× magnification, the leaf slices were placed on a glass slide in a drop of lactic acid, and the number of trichomes per 10× microscopic field was recorded. Three separate counts of trichomes were made.

### Organic acid estimation

Oxalic acid was determined by the method described by Bergerman (1955) (Bergerman, 1955) as described below.

### Preparation of indole reagent

A 100 ml of indole was dissolved in 100 ml of concentrated sulfuric acid. Every day, this reagent should be freshly made for optimal outcomes. High blank rates will result from solutions that have been prepared 24 h or more in advance of usage.

### Preparation of standard solutions

A 1 N sulfuric acid was used to dissolve oxalic acid or sodium oxalate, with concentrations ranging from 0.100 mg to 1.00 mg of oxalic acid (H_2_C_2_O_4_) per milliliter.

### Procedure

Leaf samples (0.1 g) containing oxalic acid were dissolved in a specified volume (2.0 mL) of 1 N sulfuric acid. A 2.0-mL aliquot of the each sample containing oxalic acid was taken in a test tube. For standard curve preparation, test tubes were filled with 2.0 mL of each of the pure oxalic acid standard solutions (0.100 mg to 1.00 mg of oxalic acid per mL). Then, 2.0 mL of 1 N sulfuric acid was added in the test tube to create a reagent blank. Indole reagent (2.0 mL) was added to each tube, letting it drip down the side to reduce the amount of heat that develops. After a minute, all tubes were mixed well. Then, the tubes were placed in a water bath set between 80° and 90°C for 45 min. After cooling, the absorbance was measured using a spectrophotometer at 525-nm wavelength. System was calibrated to zero by using reagent blank containing 1 N sulfuric acid and indole reagent. A standard curve was prepared using known standards and used for calculation of oxalic acid in tested samples.

Malic acid was determined by the method described by Bhagwat et al. (1995) with minor amendments (Bhagwat et al., 1995). An equal amount of leaf sample (0.03g) was taken in 15-mL screw cap tubes, and 12 mL of distilled water was added. Samples were vortexed for 2 min to wash out all acid on leaves. The water containing the acid was titrated as two subsamples of 5 mL each for acidity with 0.01 N NaOH using phenolpthlene as indicator. The mean of the two titration values, adjusted for leaf fresh weight, were then used to calculate milliequivalents of acidity for each genotype.

### Biochemical analysis

The biochemical analysis of leaf samples from resistant/tolerant and susceptible chickpea mutants/standards was performed. The mutants were characterized for different biochemical markers, i.e., antioxidants (enzymatic and non-enzymatic), oxidants (total oxidant status), and stress biomarkers (proteins, etc.) regarding insect damage (Nasir et al., 2017).

### Sample extraction

Grinded chickpea leaves (0.2 g) were extracted in potassium phosphate buffer and centrifuged for 10 min at 4°C, and the supernatant was separated and used for further assays. All data were recorded in triplicated.

### Plant pigment determination

Plant pigments including carotenoids, lycopene, chlorophyll a, chlorophyll b, as well as total chlorophyll contents were determined by a previously described method (Lichtenthaler and Wellburn, 1983).

### Enzymatic antioxidants

#### Ascorbate peroxidase activity

The ascorbate peroxidase (APX) activity of chickpea leaves sample were measured using the method of (Dixit et al., 2001). The oxidation rate of ascorbic acid was assessed by decrease in absorbance at 290 nm after every 30 s (Chen and Asada, 1989).

#### Catalase activity

Catalase (CAT) activity was determined by using the method described by (Beers and Sizer, 1952). CAT was expressed on the basis of fresh leaf weight.

#### Peroxidase activity

Activity of peroxidase (POD) was estimated using the method of (Chance and Maehly, 1955).

#### Superoxide dismutase activity

Determination of superoxide dismutase (SOD) activity was done by following the method of (Giannopolitis and Ries, 1977). One unit of SOD represented the amount of enzyme that caused 50% inhibition of photochemical reduction of NBT.

### Non-enzymatic antioxidants

#### Total phenolic content

A micro colorimetric method as previously described by (Ainsworth and Gillespie, 2007) was followed for determination of total phenolics content, using Folin–Ciocalteau reagent.

#### Total flavonoid content

An aluminum chloride (AlCl_3_) colorimetric method (Grzegorczyk et al., 2007) was followed to determine total flavonoid content (TFC) in chickpea leaves sample.

### Tannin estimation

The supernatant from total phenolic content (TPC) was not discarded; PVP.P (0.1 g) was added in TPC remaining samples, vortexed, and centrifuged; and the supernatant was used to take absorbance at 765 nm for determination of tannins.

### Ascorbic acid

For ascorbic acid, the 2,6-dichloroindophenol method previously described by (Hameed et al., 2005) was used.

### Total antioxidant capacity

A method earlier described by (Ahmed and Mohammed, 2020) was used for total antioxidant capacity (TAC) assay.

### Hydrolytic enzymes

#### Esterase activity

The α-esterases activity was estimated by earlier reported method (Van Asperen, 1962) using α–naphthyl acetate as substrate. Esterase activity was expressed as α naphthol production in µM min^−1^ per g leaf weight.

#### Protease activity

Protease activity was determined by the casein digestion assay which was previously described by (Drapeau, 1974). Enzyme activity was expressed on basis of fresh leaf weight.

#### Alpha amylase activity

The alpha amylase activity of chickpea leave samples was estimated by the modified method as reported previously (Varavinit et al., 2002).

### Other biochemical parameters

#### Total soluble proteins

Determination of total soluble protein (TSP) of chickpea leave samples were performed by earlier described method (Bradford, 1976).

#### Total oxidant status

Total oxidant status (TOS) of chickpea leave samples was determined by using earlier reported method (Erel, 2005), which is based upon the oxidation of ferrous ion to ferric ion by presence of oxidants in the sample in an acidic medium and the measuring of ferric ion by xylenol orange (Harma et al., 2005).

### Malondialdehyde content

The level of lipid peroxidation in chickpea leaves was determined in terms of malondialdehyde (MDA; a product of lipid peroxidation) content estimated by the thiobarbituric acid reaction using method of (Heath and Packer, 1968) with minor modifications as described by (Dhindsa et al., 1981).

#### Sugar contents

Reducing sugar contents in leaves was determined by the dinitrosalicylic acid method (Miller, 1959), whereas total sugars contents were determined by the phenol-sulfuric acid reagent method (Dubois et al., 1951).

### Statistical analysis

Experimental data were collected as mean ± SD. Data were the subjected to descriptive statistics for analysis. Two-way ANOVA was used with three replications for analysis of data. Tukey's Honest Significant Difference (HSD) test, p < 0.05 (where appropriate p < 0.01), was applied to test the significance of data by using the software XL-STAT. Furthermore, agglomerative hierarchical clustering (AHC) and principal component analysis (PCA) were performed using Microsoft excel as well as XL-STAT version 2012.1.02, Copyright Addinsoft 1995–2012 (http://www.xlstat.com).

## Results

### Field screening and morphological traits

#### Trichome density (hairiness) on stem and leaves

**Figure 1:**
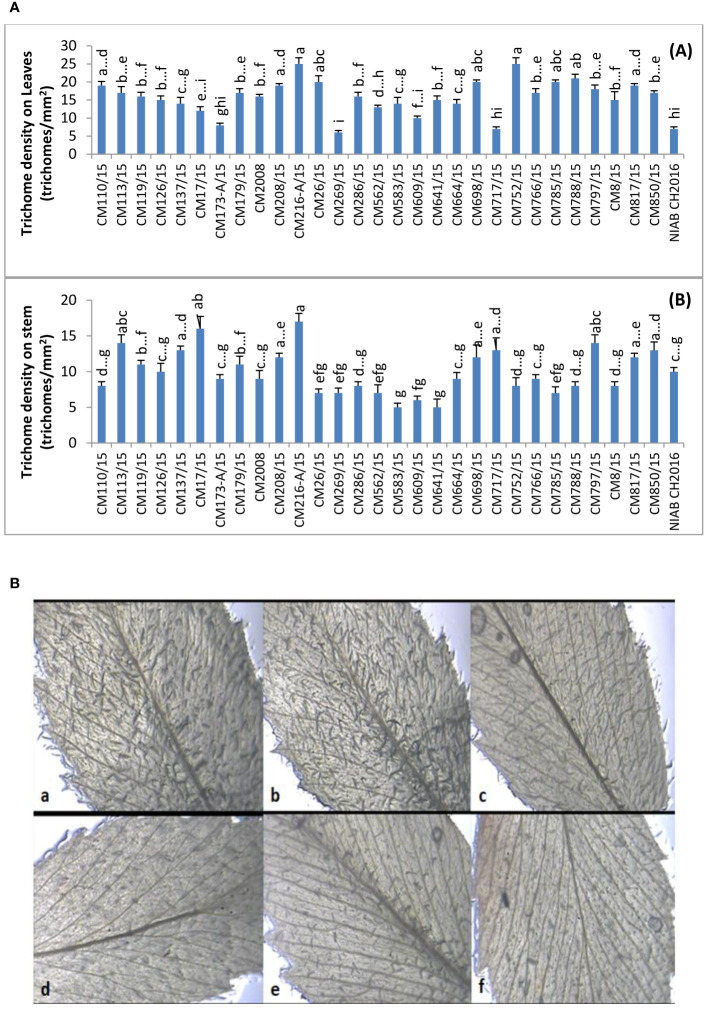
**(A)** Comparison of Trichome density (hairiness) on (A) leaves and (B) stem of chickpea genotypes. **(B)** Visual representation of trichome density (hairiness) on leaves of chickpea mutants. (a) CM216-A/15, (b) CM126/15, and (c) CM766/15 are resistant mutants, whereas (d) CM609/15 and (e) CM817/15 are sensitive mutants and (f) CM2008 is check variety.

Leaf trichome density was recorded for each chickpea genotype and expressed in trichomes/mm^2^ (**Figure 1A**). The highest leaf trichome density (25 trichomes/mm^2^) was observed in two chickpea mutants CM216-A/15 and CM752/15. On the other side, lowest leaf trichome density (06 trichomes/mm^2^) was observed in chickpea mutant CM269/15 followed by CM717/15 and standard check NIAB CH-2016. Further, a relatively lower trichome density on leaves was observed in chickpea mutants CM609/15 (10 trichomes/mm^2^) and CM583/15 (14 trichomes/mm^2^) with a sensitive response to CPB. Chickpea mutant CM216-A/15 was observed with highest trichome density on stem (17 trichomes/mm^2^) (**Figure 1A**). The lowest stem trichome density was observed in CPB-sensitive chickpea mutants, i.e., CM583/15 and CM641/15 (05 trichomes/mm^2^), followed by CM609/15 (06 trichomes/mm^2^). Representative figures showing trichome density on leaves of tolerant (CM216-A/15, CM126/15, and CM766/15) and sensitive chickpea mutants (CM609/15 and CM817/15) and check variety (CM 2008) are presented as **Figure 1B**. A clear difference in trichome density on leaves can be used to visually differentiated resistant and sensitive mutants.

### Field screening for pod borer (*Helicoverpa armigera*) at NIAB, Faisalabad

**Figure 2:**
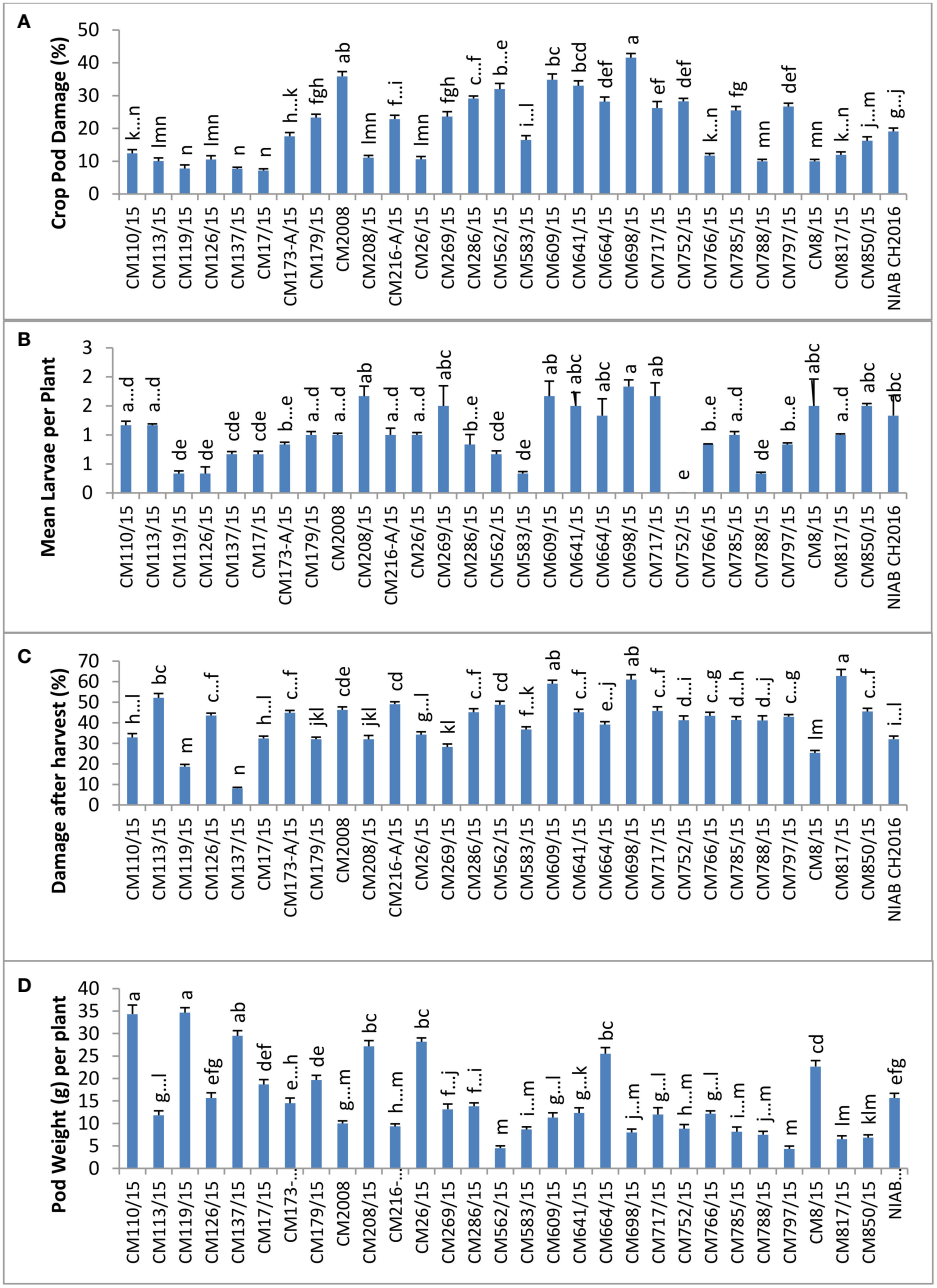
Comparison of **(A)** crop pod damage, **(B)** mean larvae per plant, **(C)** damage after Harvest, and **(D)** pod weight in chickpea mutants at Nuclear Institute of Agriculture and Biology (NIAB), Faisalabad.

CPD (%) was calculated in chickpea mutants and check genotypes (**Figure 2**), and the highest percentage was observed in CM698/15, which was 41.58 ± 12.26. Lowest percentage of CPD was observed in CM17/15 (7.163 ± 2.43). Mean larval population per plant was found maximum in CM698/15 (1.9 ± 0.78), whereas no larvae were observed in CM752/15 (**Figure 2**). Percent damage after harvest was highest in CM817/15 (92.75 ± 24), and it was in CM137/15 (38.10 ± 3.75) (**Figure 2**). Pod weight (g) per plant was found to be highest in chickpea mutant CM119/15, which was 34.67 ± 3.72 g per plant, and the least value was recorded in CM797/15 (4.33 ± 0.55 g per plant) (**Figure 2**).

### Field screening for pod borer (*Helicoverpa armigera*) at GBRS, Kallur Kot

**Figure 3:**
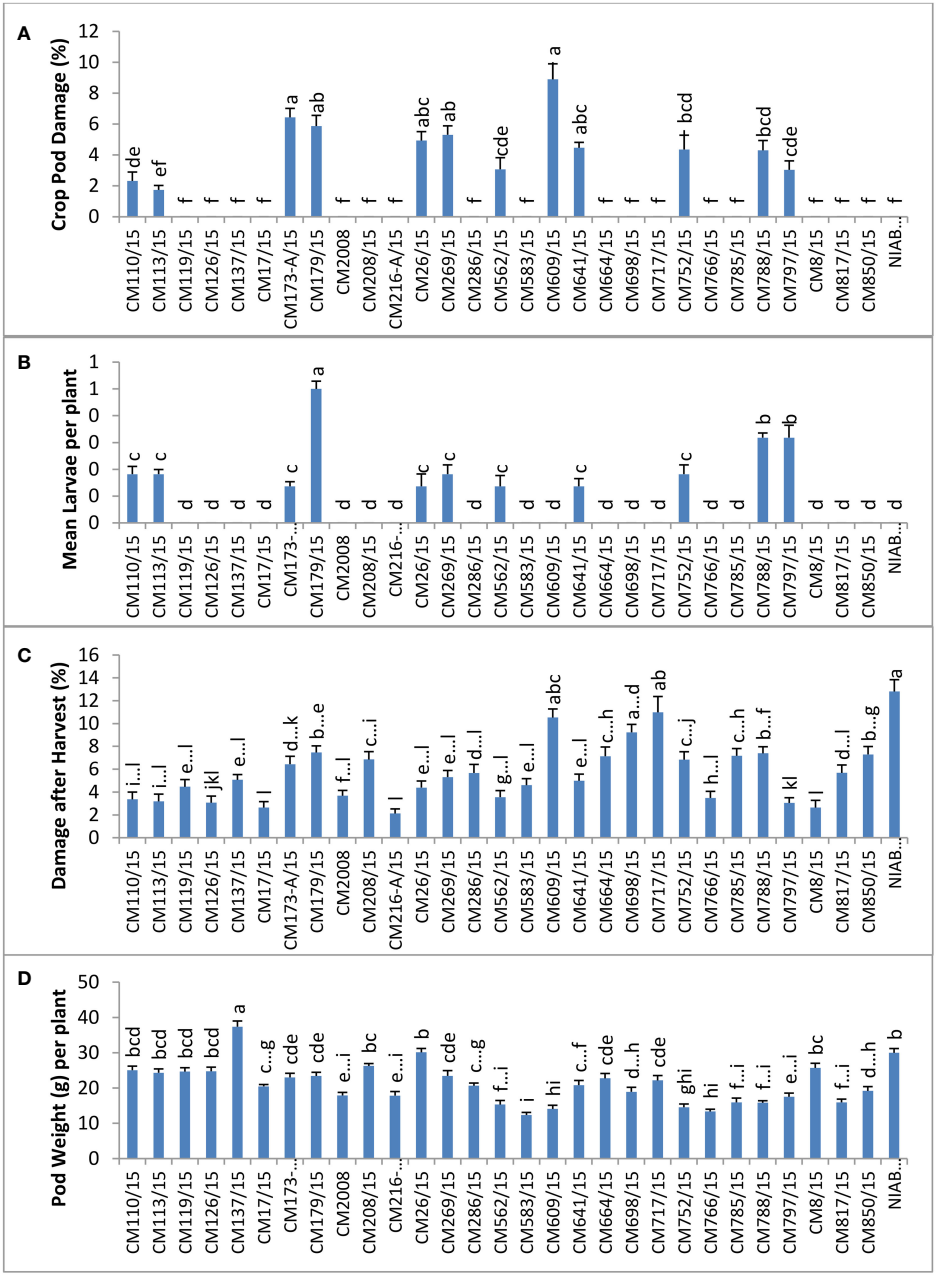
Comparison of **(A)** crop pod damage, **(B)** mean larvae per plant, **(C)** damage after harvest, and **(D)** pod weight in chickpea mutants in Kallur Kot.

At GBRS Kallur Kot, the highest percentage of CPD was observed in CM609/15 (8.90 ± 1.0), whereas a zero CPD percentage was observed in CM126/15, proving it most tolerant mutant (**Figure 3**). Mean larval population was recorded at Kallur Kot site, and the results depicted that only eleven chickpea mutants were observed with larvae, whereas no pest infestation as detected in other tested mutants (**Figure 3**). Among infested mutants, highest mean larvae per plant were observed on CM179/15 (0.5 ± 0.2) and lowest on CM641/115 (0.1 ± 0.1). The highest percentage of damage after harvest at this site was observed in NIAB CH2016 (12.8 ± 4.9), and the lowest was measured in CM216-A/15 (2.1 ± 0.7). Pod weight was observed highest in CM137/15 (37.4 ± 1.6) and lowest in CM583/15 (12.33 ± 0.7) (**Figure 3**).

### Field screening for pod borer (*Helicoverpa armigera*) at NIA, Tandojam

**Figure 4:**
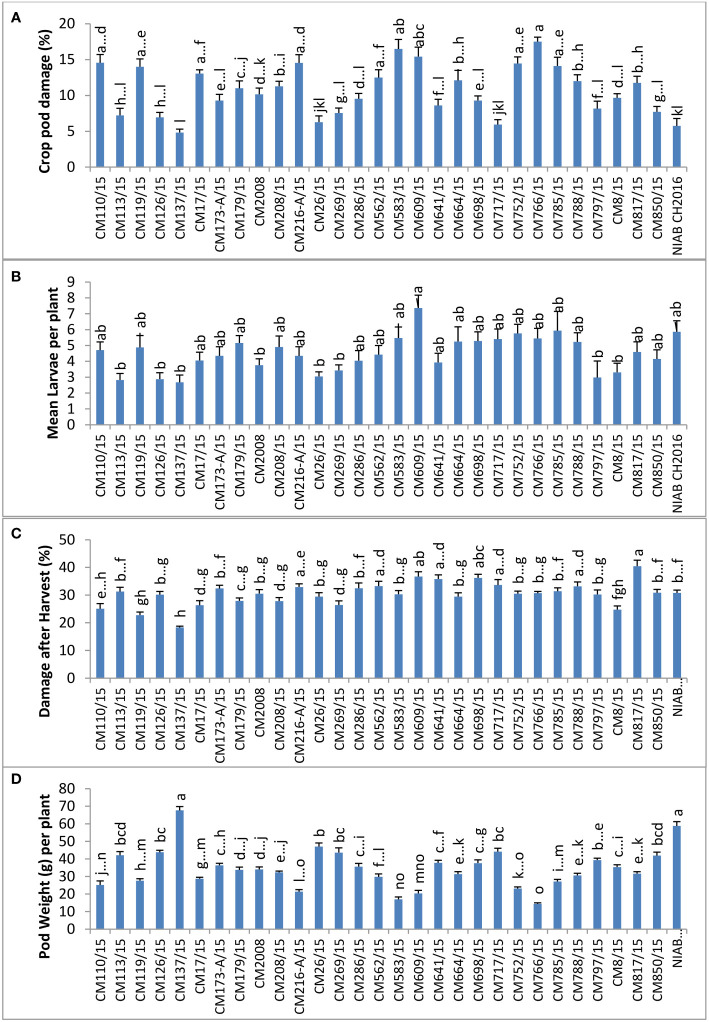
Comparison of **(A)** crop pod damage, **(B)** mean Larvae per plant, **(C)** damage after harvest, and **(D)** pod weight in chickpea mutants in Nuclear Institute of Agriculture (NIA), Tandojam.

The highest percentage of CPD was observed in CM766/15 (17.5%), and the lowest percentage was observed in CM137/15 (4.836) (**Figure 4**). The highest number of larvae per plant (7.4) was observed in CM609/15, and the least number of larvae was observed on CM137/15 (2.7). The highest percentage of damage after harvest at this site was observed in CM817/15 (40.5 ± 2.1), and the lowest percentage was measured in CM137/15 (18.2 ± 0.4).CM137/15 showed highest pod weight, which was 67.6 g per plant, whereas CM766/15 showed lowest that was 14.4 g per plant (**Figure 4**).

### Field screening for pod borer (*Helicoverpa armigera*) at AZRI, Bhakkar

**Figure 5:**
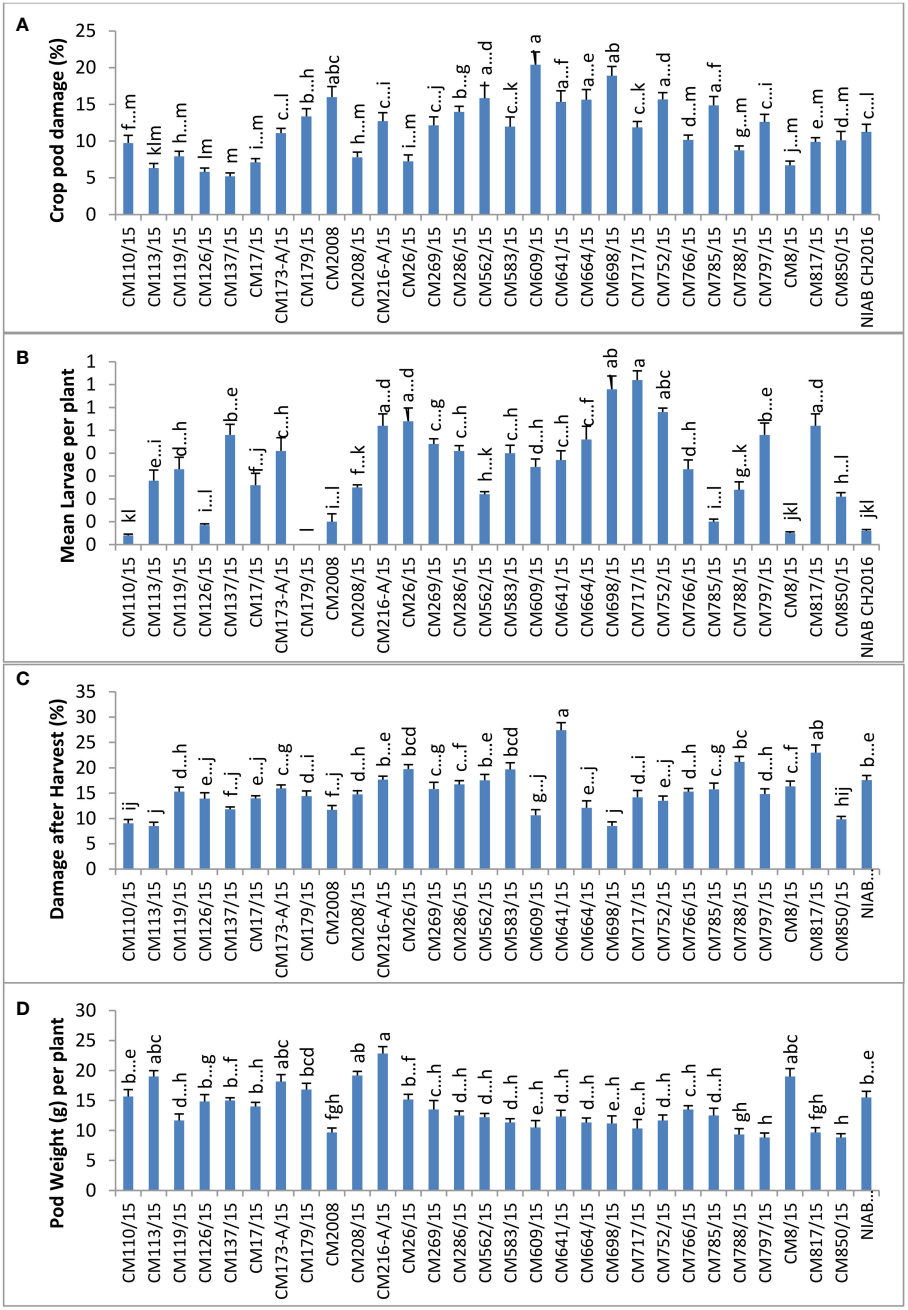
Comparison of **(A)** crop pod damage, **(B)** mean larvae per plant, **(C)** damage after harvest, and **(D)** pod weight in chickpea mutants at Arid Zone Research Institute (AZRI), Bhakkar.

At AZRI, Bhakkar, the highest percentage of CPD was observed in CM609/15 (20.4%), and the lowest percentage was observed in CM137/15 (5.2%). Maximum mean larvae per plant were observed in CM717/15 (0.72 ± 0.2), whereas no larvae were observed in CM179/15 (**Figure 5**). The highest percentage of damage after harvest was observed in CM641/15, which was 27.4 ± 3.0, whereas the lowest percentage was observed in CM113/15 (8.5 ± 0.3). CM216-A/15 showed highest pod weight (22.8 ± 2.6 g per plant) at this site, whereas the least pod weight was noticed in CM850/15 (8.8 ± 0.1 g per plant) (**Figure 5**).

### Metabolic and biochemical analysis

Multiple biochemical markers were applied to find biochemical indices of resistance/tolerance of CPB. Considerable variation was detected in biochemical profiles and stress biomarkers allowing finding their association with CPB resistance traits in chickpea.

### Organic acid estimation

**Figure 6:**
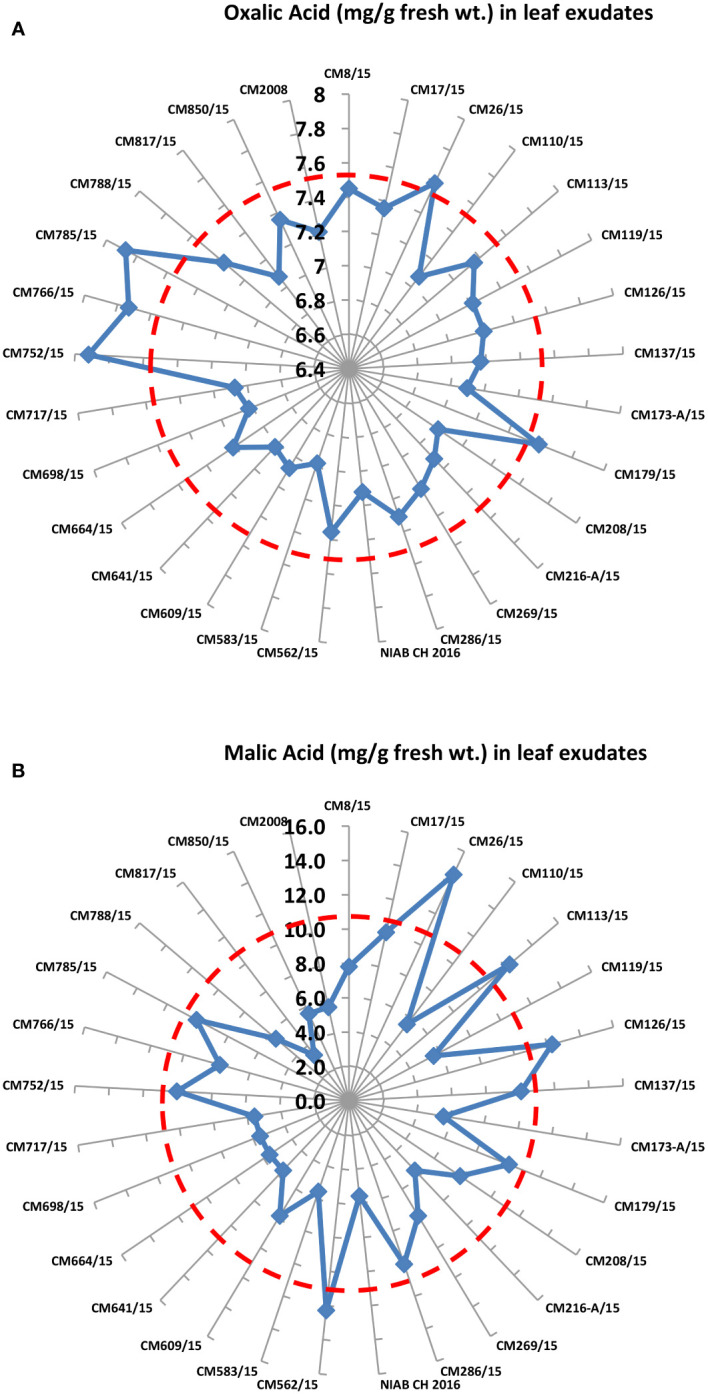
Comparison of **(A)** Oxalic acid and **(B)** malic acid in 30 chickpea mutants.

Levels of oxalic acid and malic acid were determined in 30 chickpea mutants. Maximum oxalic acid was observed in CM752/15, i.e., 7.92 mg/g F. wt., whereas the least was observed in CM583/15, which is 6.98 mg/g F. wt. (**Figure 6**). Maximum malic acid was observed in CM26/15, i.e., 14.5 mg/g F. wt., whereas the least was observed in CM817/15, which is 3.4 mg/g F. wt. (**Figure 6**).

### Plant pigments

**Figure 7:**
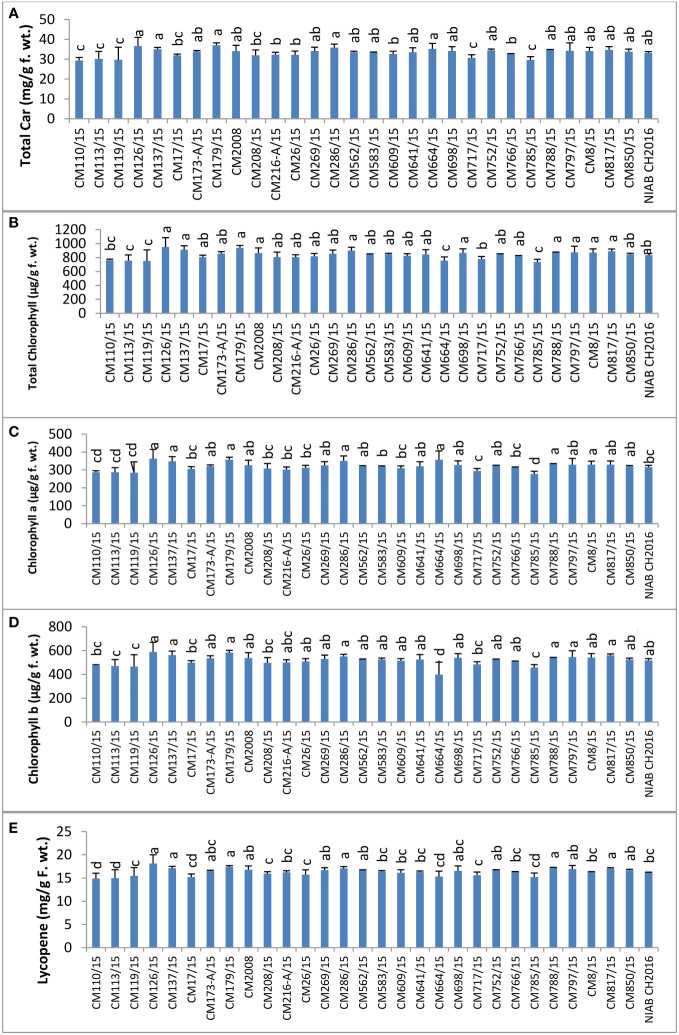
Comparison of **(A)** total carotenoids, **(B)** total chlorophyll, **(C)** chlorophyll a, **(D)** chlorophyll b, and **(E)** lycopene in chickpea mutants.

Total carotenoids were determined in plant leaves of selected chickpea mutants (**Figure 7**). Total carotenoid content was observed highest in CM179/15 (37.13 ± 1.5 mg/g F. wt.). The lowest level was observed in CM110/15, i.e., 29.44 ± 0.67 mg/g F. wt. Maximum chlorophyll was observed in CM126/15, which was 950.74 ± 14.48 µg/g F. wt. (**Figure 7**). The lowest level was observed in CM785/15, i.e., 733.93 ± 25 µg/g F. wt. CM126/15 showed maximum chlorophyll a, which was 362.70 ± 8.49 µg/g F. wt. (**Figure 7**). The lowest level was observed in CM785/15, i.e., 276.96 ± 10.26 µg/g F. wt. Again, CM126/15 showed maximum chlorophyll b, which was 588.04 ± 5.99 µg/g F. wt. (**Figure 7**). The lowest level was observed in CM664/15, i.e., 398.36 ± 14.76 µg/g F. wt. The maximum amount of lycopene was observed in CM126/15, which was 18.12 ± 1.89 mg/g F. wt. (**Figure 7**). The lowest level was observed in CM110/15, i.e., 14.90 ± 1.15 mg/g F. wt. Sensitive chickpea mutants, i.e., CM817/15, CM850/15, and CM583/15, showed higher lycopene levels as compared to tolerant chickpea mutants, i.e., CM664/15 CM216-A/15, and CM766/15.

### Enzymatic antioxidants

**Figure 8:**
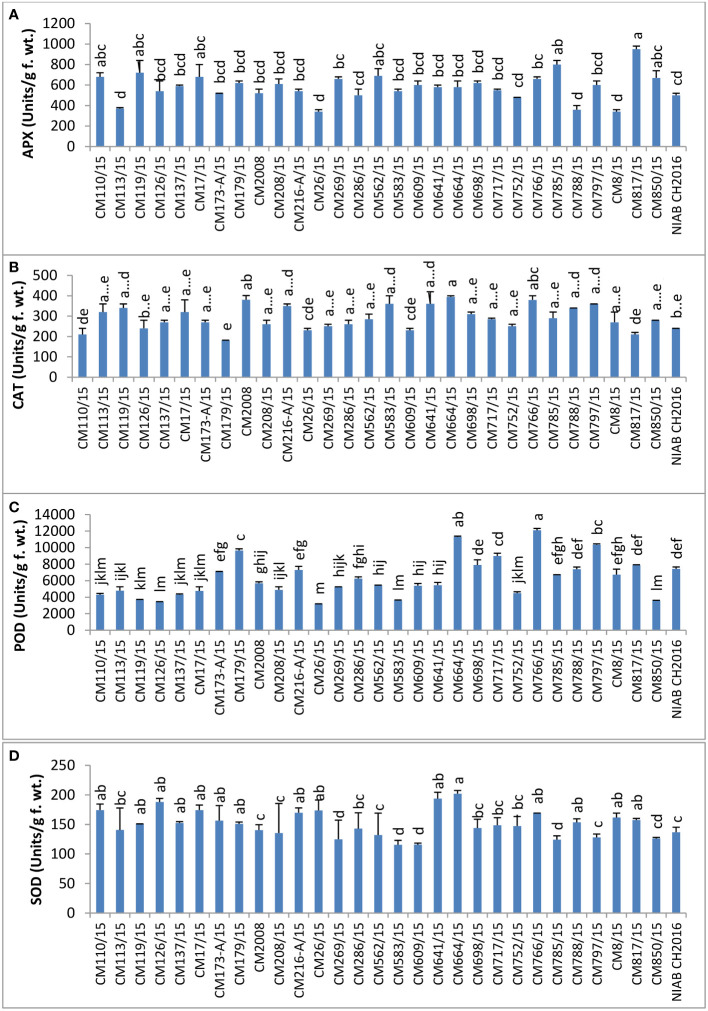
Comparison of **(A)** ascorbate peroxidase (APX), **(B)** catalase (CAT), **(C)** peroxidase (POD), and **(D)** superoxide dismutase (SOD) activities in chickpea mutants.

APX activity was tested in leaves of chickpea mutants and found highest in CM817/15 (950 ± 40 units/g F. wt.) whereas it was least in mutants CM26/15 and CM8/15, i.e., 340 ± 20 units/g F. wt. (**Figure 8**).

Catalase activity was highest in CM664/15 (395 ± 5.00 units/g F. wt.) but lowest in CM179/15, i.e., 181 ± 1.00 (**Figure 8**). Catalase activity was found to be comparatively higher in tolerant chickpea mutants i.e.CM664/15, CM766/15, and CM216-a/15, as compared to sensitive chickpea mutants, i.e., CM817/15 and CM609/15.

POD activity was highest in CM766/15 (12089.40 ± 164.50 units/g F. wt.), whereas CM26/15 showed lowest activity, which was 3162 ± 234 units/g F. wt. (**Figure 8**). POD was observed higher in tolerant chickpea mutants, i.e., CM664/15 and CM766/15, as compared to sensitive chickpea mutants like CM850/15 and CM583/15.

SOD activity was tested in the chickpea leaves, and the highest activity was measured in CM664/15, which was 202.07 ± 5.39 units/g F. wt. (**Figure 8**). CM583/15 had lowest SOD activity that was 115.496 ± 7.54 units/g F. wt. In general, SOD activity was comparatively higher in tolerant chickpea mutants, i.e., CM664/15 and CM766/15 as compared to sensitive mutants, i.e., CM583/15 and CM609/15.

### Non-enzymatic antioxidants

**Figure 9:**
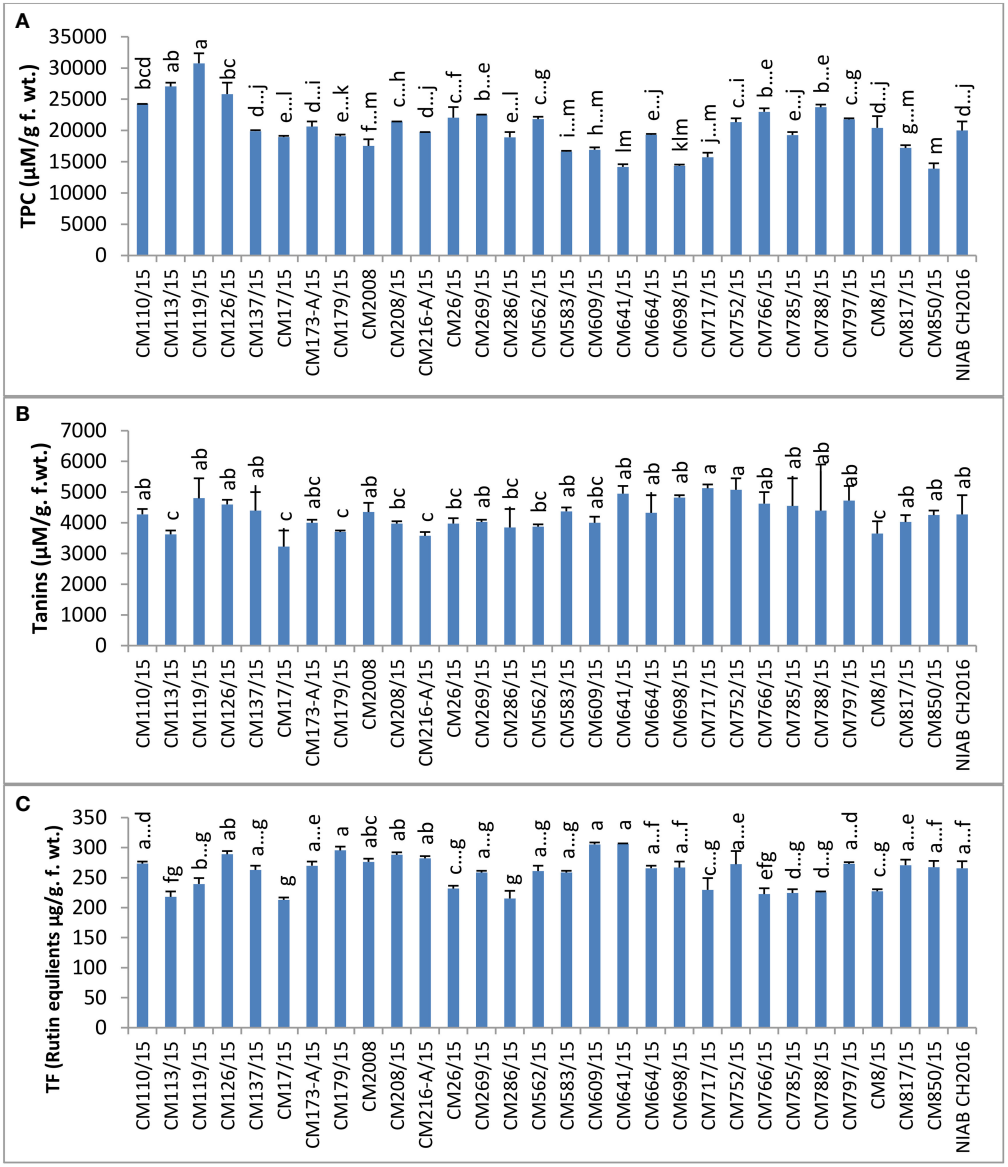
Comparison of **(A)** total phenolic contents (TPC), **(B)** tannins, and **(C)** total flavonoids (rutin equlients) in chickpea mutants.

Among non-enzymatic antioxidants, TPC was determined in the leaves of chickpea mutants and found maximum in CM119/15 (30750.0 ± 0 units/g F. wt.) (**Figure 9**). The lowest level of TPC was observed in CM850/15, i.e., 13900. ± 1450 units/g F. wt. Sensitive chickpea mutants CM850/15, CM583/15, CM609/15, and CM817/15 showed lower TPC levels than tolerant chickpea mutants, i.e., CM766/15, CM664/15, and CM216-A/15. Tannin content ranged from a highest value of 5125 ± 18 µM/g F. wt. in CM717/15 to a lowest value of 3225 ± 63 µM/g F. wt. in CM17/15. Total flavonoid (TF) value was highest in CM641/15, which was 306.205 ± 3.45 µg/g F. wt., and it was lowest in CM17/15 (212.916 ± 11.66 µg/g F. wt.) (**Figure 9**).

**Figure 10:**
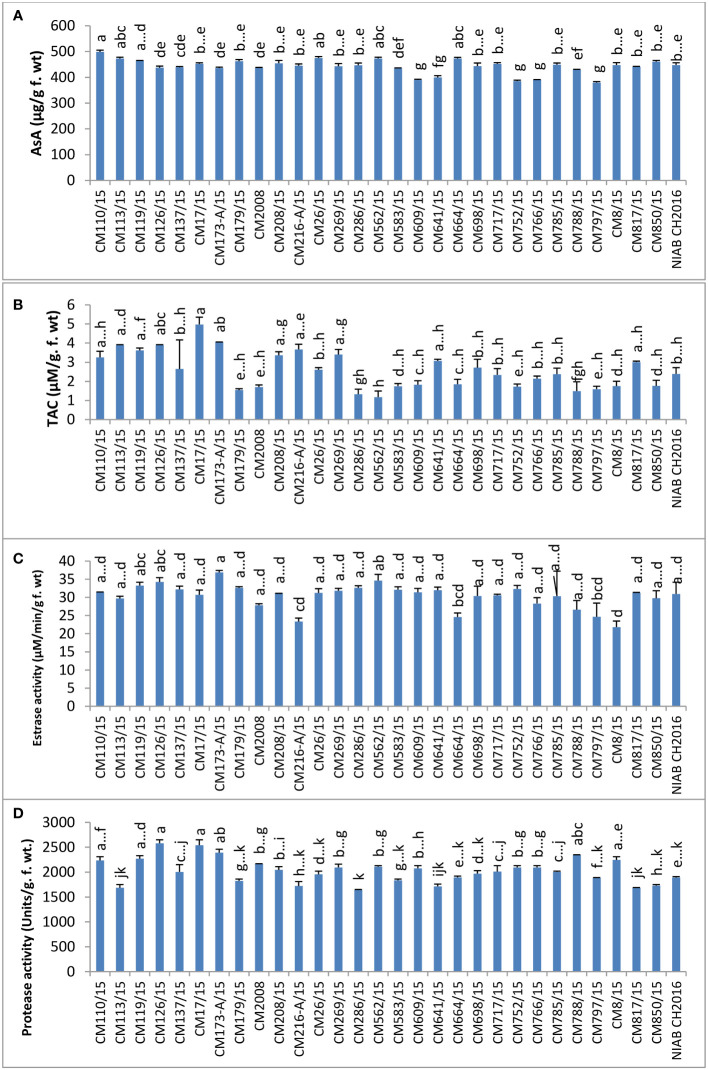
Comparison of **(A)** ascorbic acid (AsA), **(B)** total antioxidant capacity (TAC), **(C)** esterase, and **(D)** protease activities in chickpea mutants.

Ascorbic acid (AsA) was found highest in mutant CM110/15 (499.00 ± 6.5 µg/g F. wt.) and lowest in CM797/15, which was 379.750 ± 8.750 µg/g F. wt. No specific trend was observed in levels of ascorbic acid in sensitive and tolerant chickpea mutants (**Figure 10**).

TAC was determined in chickpea mutants and found highest in CM17/15, which was 4.98 ± 0.32 µM/g F. wt. CM562/15 showed the least level of TAC (1.174 ± 0.330 µM/g F. wt.). The higher level of TAC was detected in tolerant chickpea mutants, i.e., CM216-A/15, CM664/15, and CM664/15, as compared to sensitive ones, i.e., CM583/15, CM850/15, and CM609/15 (**Figure 10**).

### Hydrolytic enzymes

Esterase activity was found highest in CM173-A/15, which was 36.88 ± 0.55 µM/min/g F. wt. and minimum in CM8/15 (21.82 ± 1.66 µM/min/g F. wt.). It was interesting to note that tolerant chickpea mutants, i.e., CM216-A/15, CM664/15, and CM766/15, displayed lower esterase activity as compared to sensitive chickpea mutants, i.e., CM583/15, CM609/15, CM817/15, and CM850/15 (**Figure 10**). Protease activity was highest in CM126/15 (2580.0 ± 75.0 units/g F. wt.) but lowest in CM286/15, i.e., 1640.00 ± 20.0 units/g F. wt. (**Figure 10**).

**Figure 11:**
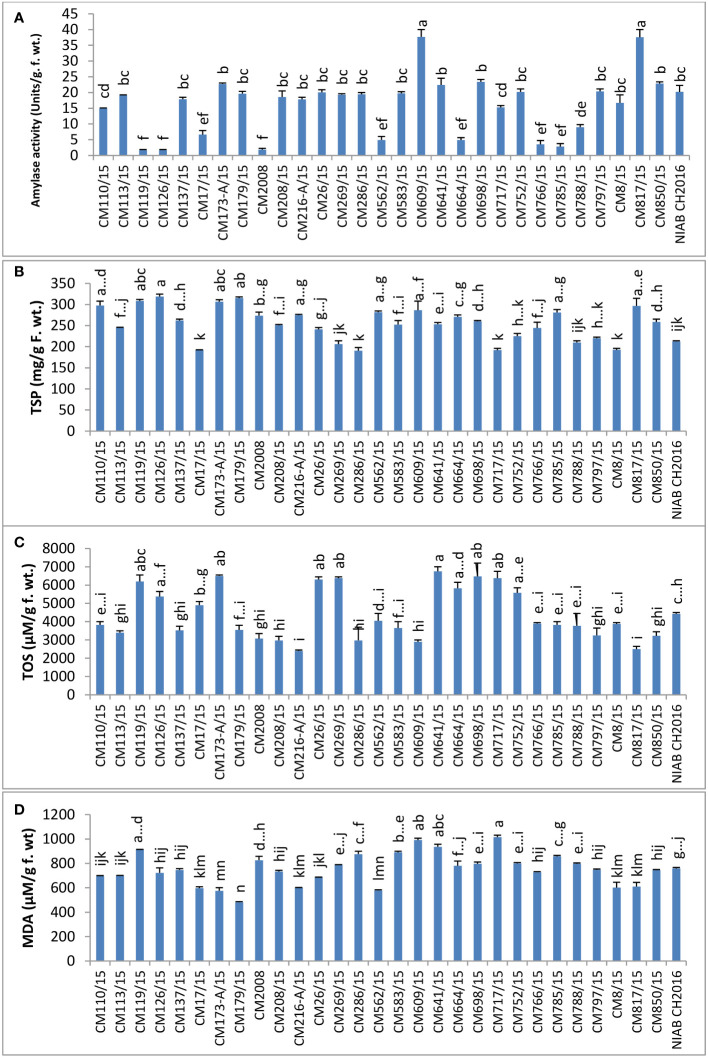
Comparison of **(A)** amylase, **(B)** total soluble proteins (TSP), **(C)** total oxidant status (TOS), and **(D)** melondialdehyde (MDA) in chickpea mutants.

In chickpea mutant CM609/15, highest amylase activity (37.70 ± 2.30 units/g F. wt.) was detected, whereas mutant CM119/15 showed least amylase activity, i.e., 1.698 ± 0.19 units/g F. wt. Tolerant chickpea mutants, i.e., CM216-A/15, CM664/15, and CM766/15, showed lower amylase activity as compared to sensitive chickpea mutants like CM583/15, CM609/15, CM817/15, and CM850/15 (**Figure 11**).

### Stress biomarkers and metabolites

TSPs were found highest in CM126/15 (318.67 ± 10.3 mg/g F. wt.) and lowest in CM286/15, which was 190.33 ± 1.67 mg/g F. wt. (**Figure 11**).

TOS, a measure of hydrogen peroxide, was observed highest in CM698/15 (6750 ± 250 µM/g F. wt.). The lowest TOS value was observed in CM216-A/15 (2400 ± 50 µM/g F. wt.). A comparatively higher TOS level was observed in tolerant chickpea mutants, i.e., CM664/15 and CM766/15 (except CM216-A/15) but lower in sensitive chickpea mutants, i.e., CM583/15, CM850/15, CM609/15, and CM817/15 (**Figure 11**).

Melandialdehyde (MDA) level was found highest in CM717/15, which was 1014.97 ± 3.097 µM/g F. wt. Lowest MDA level was detected in CM179/15 (480.77 ± 8.52 µM/g F. wt.). Sensitive chickpea mutants, i.e., CM609/15 and CM583/15, maintained higher MDA levels as compared to sensitive chickpea mutants, i.e., CM216-A/15 and CM766/15 (**Figure 11**).

**Figure 12:**
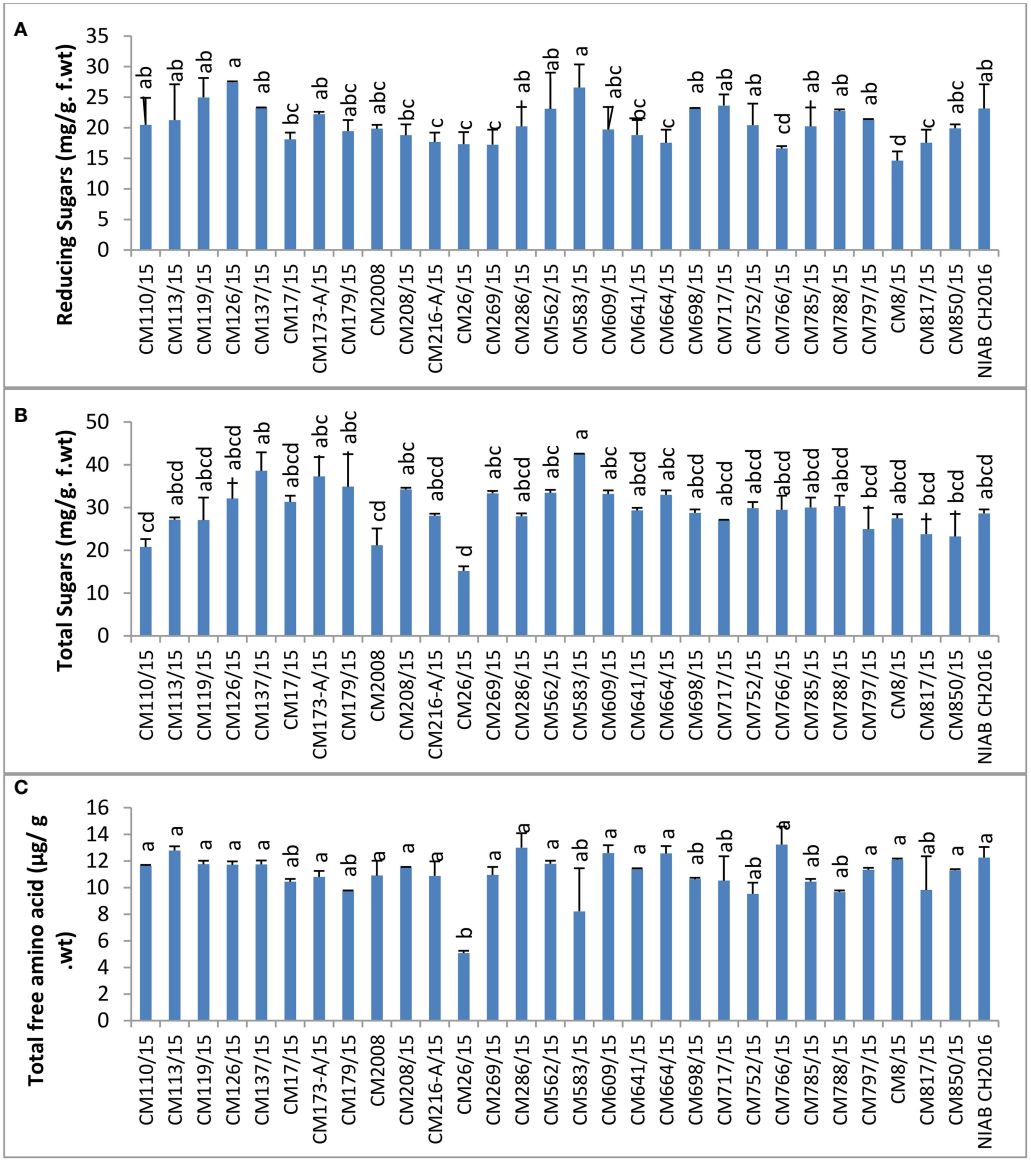
Comparison of **(A)** reducing Sugars, **(B)** total sugars, and **(C)** total free amino acids in chickpea mutants.

Reducing sugars were measured maximum in CM126/15 (42.54 ± 1.9 mg/g F. wt.) and lowest in CM26/15 (15.18 ± 0.93 mg/g F. wt.). Sensitive chickpea mutants like CM583/15, CM850, and CM609/15 showed higher level of reducing sugars as compared to tolerant chickpea mutants, i.e., CM216-A/15, CM664/15 and CM766/15, indicating a role in CPB tolerance (**Figure 12**).

The highest level of total sugars was observed in CM583/15, i.e., 42.54 ± 1.86 mg/g F. wt. The lowest total sugars were detected in CM26/15 (15.18 ± 0.93 mg/g F. wt.). A higher level of total sugars was observed in tolerant chickpea mutants, i.e., CM664/15 and CM766/15, as compared to sensitive mutants, i.e., CM817/15 and CM850/15 (**Figure 12**). Similarly, the maximum amount of total free amino acids was detected in CM766/15 (13.23 ± 0.08 µg/g F. wt.), and a least value was detected in CM26/15, which was 5.08 ± 0.800 µg/g F. wt. Again, tolerant chickpea mutants, i.e., CM766/15 and CM664/15, maintained a higher level of free amino acids as compared to sensitive mutants, i.e., CM583/15 and CM817/15 (**Figure 12**).

### Principal component analysis

**Figure 13:**
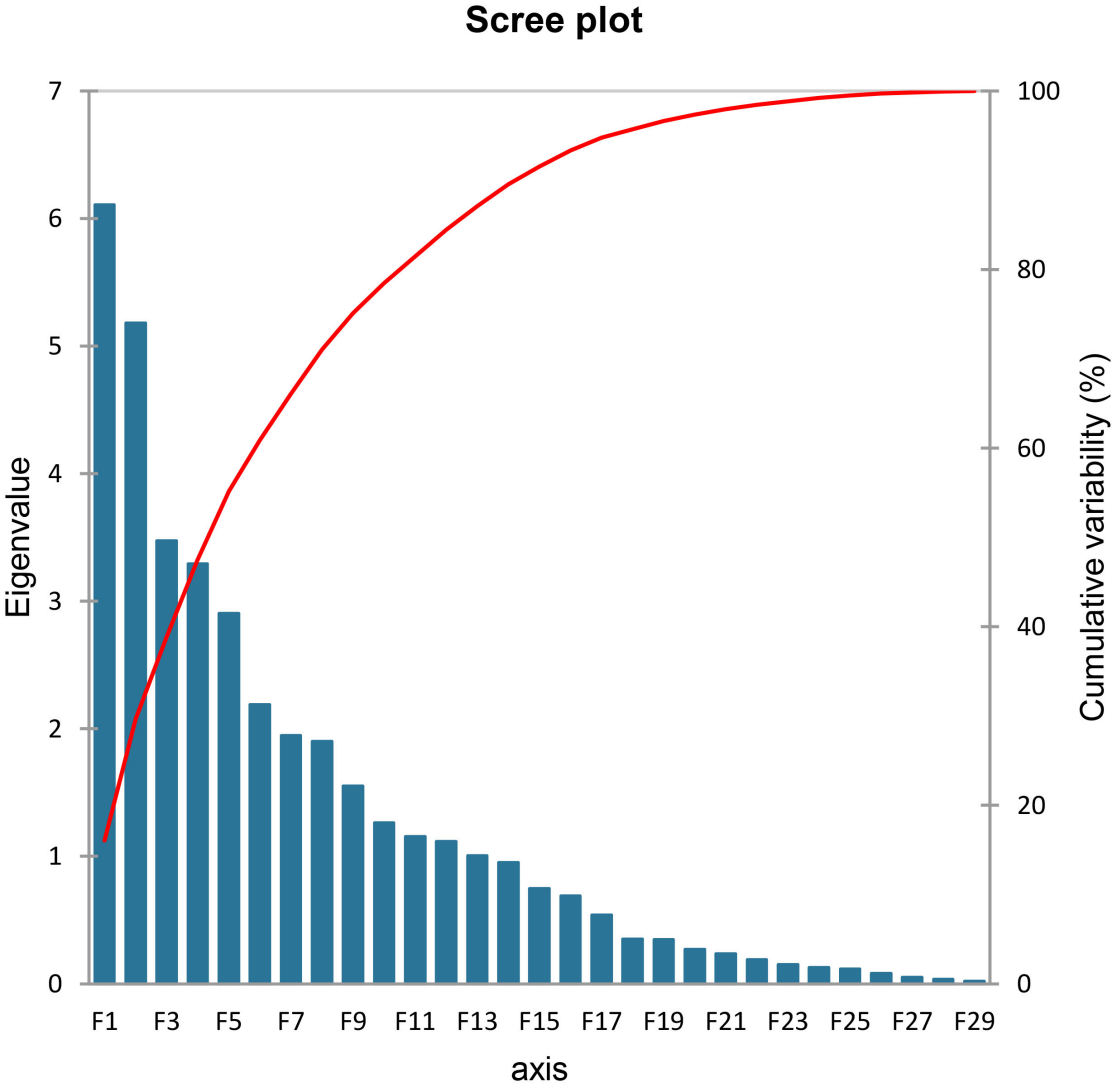
Scree plot representing cumulative variability and eigenvalues for the studied parameters.

PCA was performed on the experimental data collected from biochemical analysis. In principal components, eigenvalue > 1 best designates the system traits (Kumar et al., 2019). Eigenvalues are graphically presented in scree plot (**Figure 13**) showing total 29 principal components that are responsible for the variation in data. Thirteen of these, represented as PC-I to PC-XIII, had eigenvalues greater than 1 and acquired a major portion, i.e., 87.07% of the total collective variability. Almost 29.69% of the total variability is contributed by PC-I and PC-II, therefore called chief contributors. More than 55% of the total collective variability is because of PC-I to PC-V, whereas main source of variability is PC-I that describes maximum 16.07% variation in genetic resources (**Table 3**).

**Table 3:** Eigenvalues for the first 20 factors.

	F1	F2	F3	F4	F5	F6	F7	F8	F9	F10	F11	F12	F13	F14	F15	F16	F17	F18	F19	F20
**Eigenvalue**	6.106	5.179	3.474	3.293	2.903	2.190	1.948	1.901	1.552	1.262	1.157	1.118	1.004	0.950	0.747	0.691	0.541	0.352	0.347	0.272
**Variability (%)**	16.068	13.630	9.141	8.667	7.639	5.764	5.127	5.003	4.084	3.320	3.044	2.941	2.643	2.501	1.965	1.820	1.423	0.927	0.912	0.716
**Cumulative %**	16.068	29.698	38.839	47.506	55.146	60.910	66.037	71.040	75.124	78.444	81.488	84.429	87.073	89.574	91.539	93.359	94.782	95.709	96.621	97.337

F1 to F20: Factors (principal component of variability) from 1 to 20.

**Figure 14:**
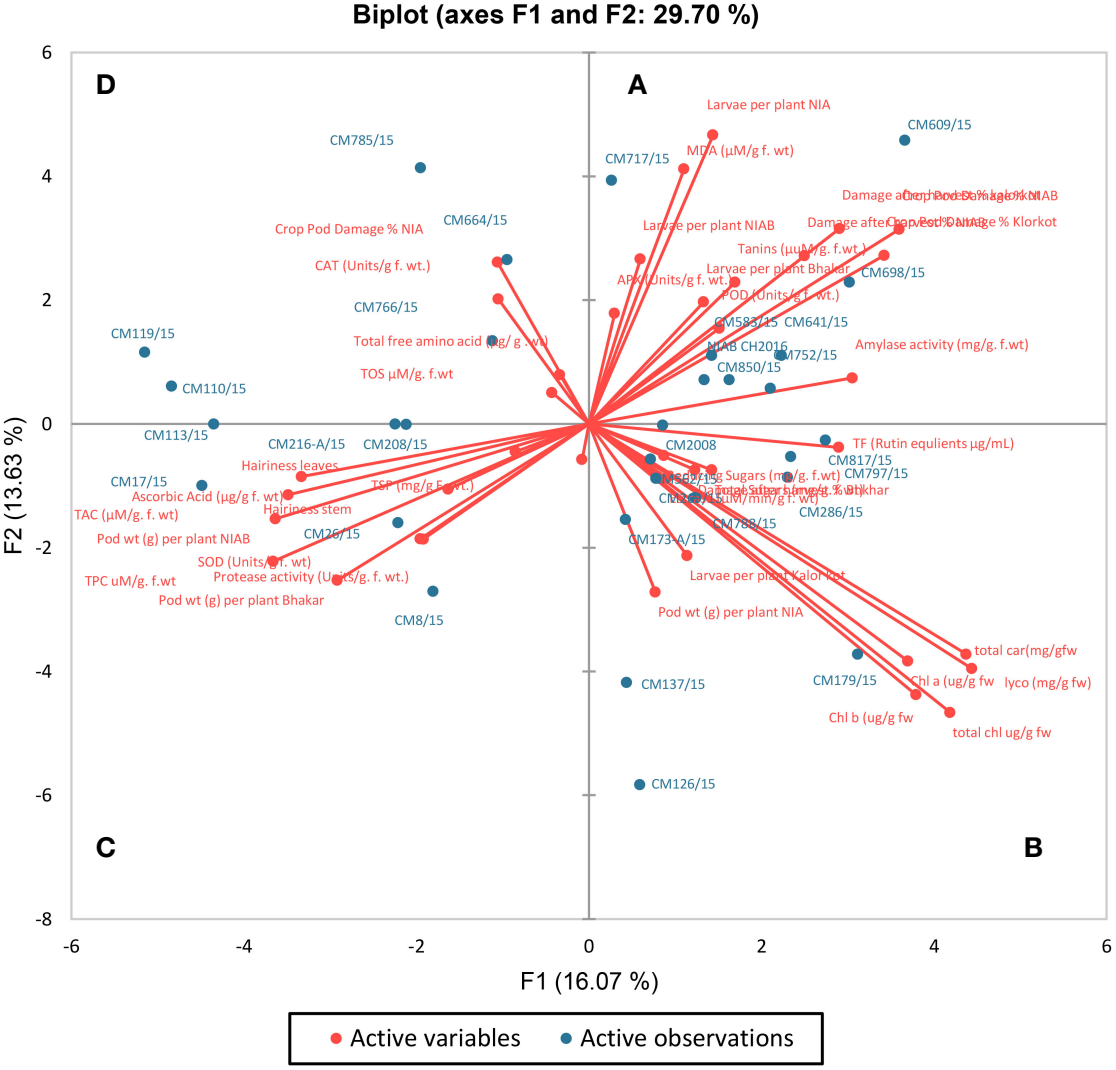
Bi-plot of chickpea genotypes for the first two principal components. Based on the angle between the traits, the biplot was categorized into four groups **(A–D)**.

For all the chickpea mutants and their respective traits, a G-T (genotype-by-trait) biplot was drawn by placing PC-I scores on the x-axis, whereas PC-II scores on the y-axis (**Figure 14**). This G-T biplot remarkably showed and explained the interrelationships among traits and genotypes/mutants and also provided a visual comparison between different chickpea mutants. Right angle (90°) was considered to be reflecting that two traits/variables are totally independent of each other (Shah et al., 2020). Four categories/groups were formed in the G-T biplot on the basis of angles between the vectors. These groups were names as A, B, C, and D. A positive correlation was shown in group A by MDA, APX, POD, amylase activity, tannins, and larvae per plant in NIAB, NIA, and Bhakkar; a positive correlation in group B was shown by total carotenoids, total chlorophyll, Chl a, Chl b, total sugars, TFs, esterase activity, lycopene, and reducing sugars; a positive correlation in group C was shown by ascorbic acid, TAC, TPC, SOD, protease activity, TSPs, hairiness of stem and leaves, and pod weight; whereas, in group D, CAT, TOS, and total free amino acids showed a positive correlation with CPD (%).

Positive factor loadings for POD, APX, lycopene, total carotenoids, chlorophyll a and b, esterase activity, amylase activity, reducing sugar, total sugars, tannins, MDA, and TFs were shown by PC-I, whereas positive factor loading for CAT, POD, APX, amylase activity, tannins, MDA, TOS, and total free amino acids was shown by PC-II (**Table 4**). Traits that contributed positive factor loadings toward PC-III were esterase activity, amylase activity, TSPs, ascorbic acid, protease activity, TAC, reducing sugars, tannins, total sugars, MDA, TFs, and TOS, whereas PC-IV contributed positive factor loadings for POD, total carotenoids, amylase activity, ascorbic acid, TAC, SOD, total free amino acids, and hairiness of stem and leaves. One variable is typically selected from these known clusters subject to individual loadings (Mishra et al., 2015). Therefore, lycopene showed the highest contribution to PC-I with factor loading score of 0.722 followed by total carotenoids with score 0.711, whereas major contributor of PC-II was total chlorophyll with factor loading 0.698. It is transpired from these results that PCA pointed out specific traits for use in breeding programs as choice of interest.

**Table 4:** Factor loadings for the tested parameters.

	F1	F2	F3	F4	F5
Catalase (CAT) (Units/g F. wt.)	−0.171	0.302	−0.472	−0.207	−0.529
Peroxidase (POD) (Units/g F. wt.)	0.246	0.231	−0.404	0.220	−0.165
Ascorbate peroxidase (APX) (Units/g F. wt.)	0.049	0.268	−0.011	−0.137	0.467
Lycopene (mg/g F. wt.)	0.722	−0.592	−0.143	−0.178	−0.017
Chlorophyll a (µg/g F. wt.)	0.601	−0.574	−0.100	−0.008	−0.150
Chlorophyll b (µg/g F. wt.)	0.616	−0.655	−0.035	−0.044	0.082
Total carotenoids (mg/F. wt.)	0.711	−0.557	−0.158	0.007	−0.084
Total chlorophyll µg/g F. wt.)	0.681	−0.698	−0.065	−0.035	−0.001
Esterase (µM/min/g F. wt.)	0.147	−0.131	0.601	−0.440	0.358
Amylase activity (mg/g F. wt.)	0.497	0.111	0.191	0.492	0.297
Total Phenolic Content (TPC) (µM/g F. wt.)	−0.595	−0.332	−0.009	−0.353	0.018
Total soluble protein (TSP) (mg/g F. wt.)	−0.013	−0.086	0.044	−0.300	0.645
Ascorbic acid (µg/g F. wt.)	−0.542	−0.127	0.321	0.243	0.217
Protease activity (units/g F. wt.)	−0.318	−0.278	0.138	−0.525	−0.011
Total antioxidant activity (TAC) (µM/g F. wt.)	−0.567	−0.171	0.292	0.043	0.134
Reducing sugars (mg/g F. wt.)	0.141	−0.077	0.394	−0.612	−0.056
Tanins (µM/g F. wt.)	0.276	0.343	0.154	−0.494	−0.435
Superoxide dismutase (SOD) (units/g F. wt.)	−0.312	−0.279	−0.120	0.030	−0.225
Total sugars (mg/g F. wt.)	0.232	−0.111	0.114	−0.368	0.198
Melandialdehyde (MDA) (µM/g F. wt.)	0.179	0.617	0.260	−0.320	−0.395
Total flavonoids (TF) (Rutin equlients µg/g F. wt.)	0.471	−0.057	0.101	−0.020	0.427
Total oxidant status (TOS) (µM/g F. wt.)	−0.070	0.076	0.506	−0.265	−0.419
Total free amino acid (µg/g F. wt.)	−0.055	0.119	−0.054	0.056	0.037
Trichome density (hairiness) on stem (trichomes/mm^2^)	−0.265	−0.158	−0.097	0.423	−0.043
Trichome density (hairiness) on leaves (trichomes/mm^2^)	−0.138	−0.067	−0.577	0.024	0.065
Crop pod damage (%) at NIAB	0.585	0.471	−0.064	0.036	−0.056
Larvae per plant at NIAB	0.097	0.399	0.283	0.691	0.033
Damage after harvest (%) at NIAB	0.407	0.407	−0.300	0.158	0.166
Pod weight (g) per plant at NIAB	−0.591	−0.230	0.380	0.000	0.061
Crop pod damage (%) at Kallur Kot	0.557	0.408	0.387	0.164	0.246
Larvae per plant at Kallur Kot	0.185	−0.319	−0.120	−0.004	0.107
Damage after harvest (%) at Kallur Kot	0.472	0.473	0.518	0.094	0.048
Crop pod damage (%) at NIA	−0.172	0.391	−0.580	−0.422	0.446
Larvae per plant at NIA	0.234	0.699	−0.012	−0.251	0.368
Pod weight (g) per plant at NIA	0.125	−0.407	0.643	0.350	−0.368
Larvae per plant at AZRI Bhakkar	0.217	0.295	0.051	0.096	−0.409
Damage after harvest (%) AZRI Bhakkar	0.200	−0.111	−0.173	−0.114	−0.132
Pod wt. (g) per plant at AZRI Bhakkar	−0.474	−0.379	0.018	0.355	0.271

NIAB, Nuclear Institute for Agriculture and Biology; AZRI, Arid Zone Research Institute; NIA, Nuclear Institute for Agriculture. F1 to F5: First five factors (principal component) with highest variability.

### Correlation analysis

Pearson test (correlation) was performed for all studied biochemical traits with 95% confidence interval. A significantly negative correlation of lycopene was observed with ascorbic acid, whereas significantly positive correlation with chlorophyll and carotenoids (**Supplementary Table 1**). Total chlorophyll exhibited significant positive correlation with other pigments. A significant positive correlation was presented by chlorophyll b with total carotenoids and total chlorophyll, whereas negative correlation with CAT. In the same way, total carotenoids were positively correlated with chlorophyll and negatively correlated with TAC. Esterase was found to be positively correlated with reducing sugars and TSP, whereas negatively correlated with CAT and POD. Amylase showed negative correlation with CAT, TPC, and protease, whereas positive correlation with TFs. TPC and TSP were positively correlated with protease and TFs, respectively. Tannins were positively correlated with reducing sugars, whereas negatively with MDA and TOS.

### Cluster analysis

**Figure 15:**
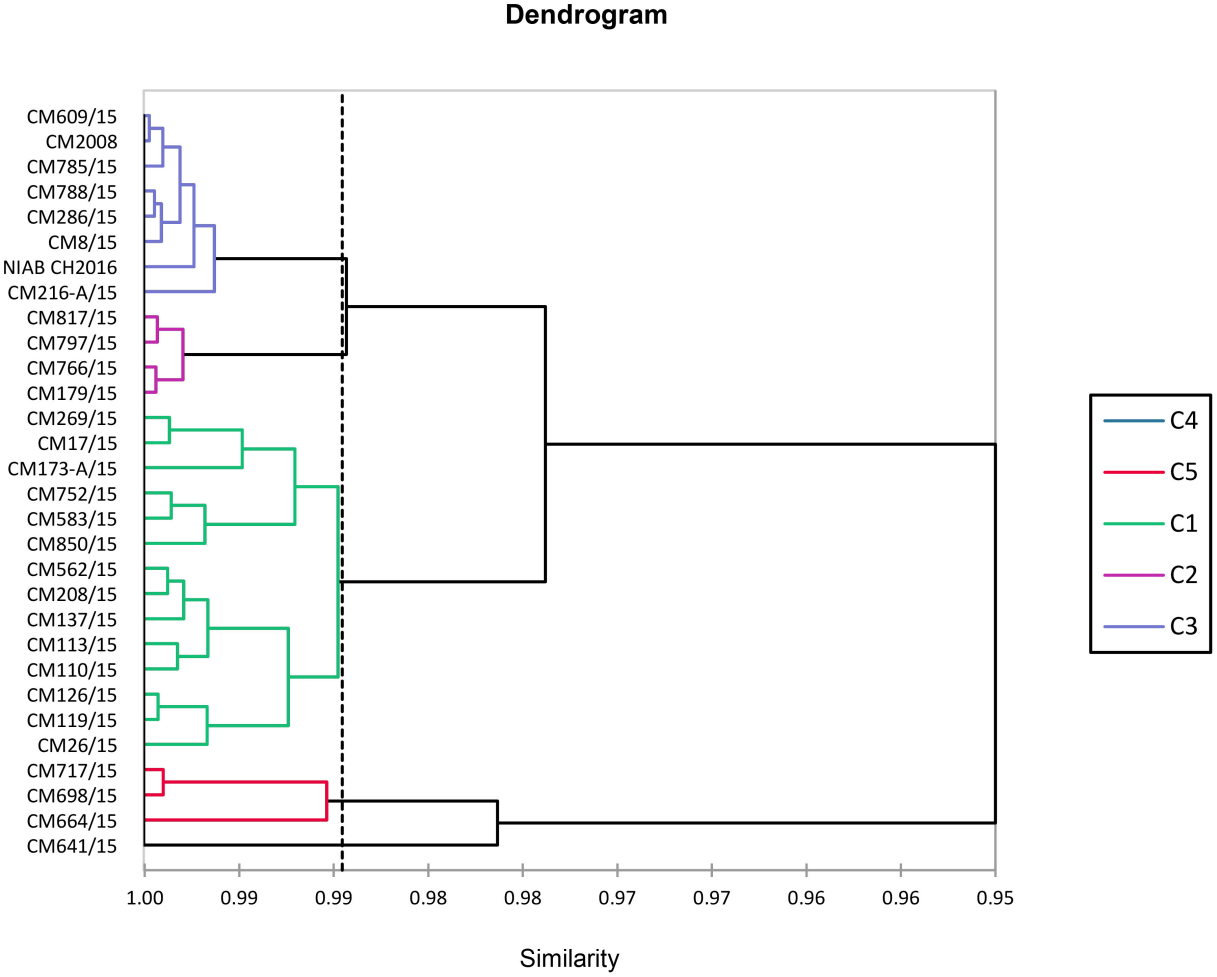
Cluster diagram showing grouping of chickpea mutants.

AHC of chickpea mutants based on studied traits is shown in **Figure 15**. All 30 chickpea mutants were grouped into five clusters by cluster analysis. Cluster-I consist of 14 chickpea mutants followed by four, eight, one, and three chickpea mutants correspondingly in cluster-II, cluster-III, cluster-IV, and cluster-V. Minimum variety for traits was shown in cluster-I. Among tested mutants, CM641/15 grouped separately in cluster-IV and was most diverse. Mutants of cluster-IV with maximum diversity were found suitable for making crosses against mutants of cluster-III. Chickpea mutant CM216-A/15, exhibiting high levels of resistance, was placed in cluster-III, whereas CM752/15 and CM113/15 were placed in cluster-I.

## Discussion

In comparison to cultivated chickpea, the wild relatives showed significantly higher stages of antibiosis to *H. armigera* with respect to decreased survival pupation of larva, emergence of adult, decreased weights of larval and pupal, continued larval and pupal developmental stages, and decreased level of fecundity as reported earlier (Golla et al., 2020). Similar trend was observed in current study as chickpea mutants CM216-A/15, CM752/15, CM113/15, and CM797/15 showed a higher resistance level, whereas CM641/15 and CM269/15 showed a lower resistance in terms of hairiness in stem and leaves, respectively. At various experimental sites, CM137/15, CM17/15, and CM797/15 exhibited higher resistance levels in terms of CPD, lessened larvae survival, lesser damage after harvest, and lower pod weights against when compared to the susceptible checks, i.e., CM698/15 and CM119/15. Previous research in chickpea had similarly revealed higher antibiosis levels against *H. armigera* in rough relatives when compared to cultigen with respect to decreased survival and late developmental phases (Sharmad et al., 2005; Narayanamma et al., 2007). It was earlier studied that lower survival and pupation of larva and less pupal weights were seen in F_1_ hybrids, indicating that the antibiosis resistance mechanism was passed on to the offspring from resistant parents (Narayanamma et al., 2007). According to another finding, antibiosis appears to be the main mechanism underlying resistance to *H. armigera* in chickpea, which may be brought on by increased concentrations of plant subordinate metabolites or low dietary value (Sharmad et al., 2005).

Carotenoids are naturally occurring lipophilic pigments. A low level of total carotenoid and lycopene content was observed in CM110/15, and a high content was found CM126/15 and CM179/15. During active invasion period of *H. armigera* in antioxidative defense enzymes, non-enzymatic antioxidants assays were measured in leaves of chickpea mutants. Different mutants of the chickpea showed notable variations in biochemical composition. TPC and TFC are the principal bioactive molecules among non-enzymatic antioxidants. They may be found in all plant parts and are frequently eaten (Asif, 2015). A negative connection between proteins, phenols and larval weight, pupation, and adult emergence has been reported earlier (Golla et al., 2020). Similar trend was observed in current study as CM126/15 showed higher values of TSP, phenolic contents, and lower larval population, whereas CM717/15 showed conciliatory trend. Additionally, phenols demonstrated a favorable link with pupal period but a negative association with pupal fecundity and weight. Although, Kanchana et al. (2005) earlier reported that protein had positive link with pod damage in chickpea. These variations may result from the existence of more protease inhibitors in wild cousins than in cultivated chickpea mutants (Patankar et al., 1999; Parde et al., 2012). Reserve proteins called protease inhibitors are found in plants that prevent insects from eating and digesting the material they consume (Blanco-Labra et al., 1995). Chickpea protease inhibitors show various inhibitory activity against *H. armigera* gut proteinases (Giri et al., 1998). The antibiosis properties of protease inhibitors such as long larval growth period, lessening in larval weight, survival, and adult emergence, were observed in *H. armigera* fed on diet embedded with chickpea trypsin inhibitor (Kansal et al., 2008). Higher contents of phenol present in resistant chickpea mutants when compared to the susceptible may also contribute to cause resistance against *H. armigera* (Kaur et al., 2014). The presence of phenols content in host plant may also lead to toxicity in insects (Bhonwong et al., 2009) by mediating the transduction pathways as increasing the defensive enzyme activity, which causes oxidation of toxic constituents such as quinines (Maffei et al., 2007; Bhonwong et al., 2009). It was reported previously that total phenol contents showed significant negative relationship with % pod damage caused by *H. armigera* (Salimath et al., 2008; Sunitha et al., 2008) as also observed in current study.

TSS showed a substantial negative correlation with larval growth period and positive link with pupation and pupal weight, whereas tannin content, on the other hand, exhibited a positive association with larval weight, pupation, and emergence of adult. From these findings, we concluded that the higher amount of these components preferred better survival and development stage of *H. armigera*, leading to increased susceptibility of host plant to this pest. High phenol content and low sugar have also been documented in the resistant cultivars of pigeon pea against *H. armigera* (Sharma et al., 2009). On the conflicting, it is well recognized that tannins may act as feeding restraints and decrease the survival and development of several insects (Bernards and Båstrup-Spohr, 2008), thereby inhibiting the digestion process by precipitating proteins non-specifically, which depend upon their chemical assembly and many other factors including pH of the gut and amount of antioxidants (Galati et al., 2002; Hagerman et al., 2003). Tannins act as feeding restraints on the non-adapted insects and also act as stimulants for feeding on the adapted insects (Golla et al., 2020). There were noteworthy differences among the various chickpea mutants tested with respect to the composition of flavonoids. The negative possessions of flavonoids on the performance of insect in terms of long developmental period, increased rate of mortality, reduced survival rate, weight, and fecundity had also been detected in many insect species, including *Acyrthosiphon pisum* (Goławska et al., 2014), *Epirrita autumnata* (Valkama et al., 2005) *Mamestra configurata* (Onyilagha et al., 2004), *Trichoplusia ni* (Beninger et al., 2004), *Nipaecoccus viridis* (Lahtinen et al., 2006), and *Eriosoma lanigerum* (Ateyyat et al., 2012). However, there are numerous reports regarding negative association of flavonoids on the host plant, whereas the exact mechanism by which flavonoids modify the behavior of insects still remains unknown (Simmonds, 2003). Ascorbic acid (vitamin C), a non-enzymatic antioxidant, facilitates the movement of electrons and acts as an antioxidant by scavenging reactive oxygen species and regenerating the form of vitamin E antioxidant mechanism (Prasad and Upadhyay, 2011; McGill and Jaeschke, 2013). AsA is one of the most effective antioxidants against various stresses in plants particularly in chickpeas. Highest ascorbic acid was observed in various chickpea mutants especially CM126/15 made it more resistant against pod borer.

In living systems, enzymatic antioxidants such as SOD, CAT, APX, and POD act as the first line of defense mechanism against oxidative stress as they have the solid and rapid ability to scavenge free radicals, removing hydroxyl radical and detoxifying hydrogen peroxide and oxygen intermediates in the cell (Ighodaro and Akinloye, 2018; Kohli et al., 2019). APX is the most significant POD enzyme that helps in H_2_O_2−_ scavenging, also acts as an electron donor, and defends cell elements by eliminating reactive oxygen species (Gangwar et al., 2014). Low level of APX activity was found in CM126/15. It was found previously that a higher level of APX activity was found in leaves of resistant chickpea mutants as compared to susceptible chickpea mutants that make them more resistant toward *Helicoverpa armigera* (Kaur et al., 2017). APX decreases excessive H_2_O_2_ to water by employing ascorbic acid as the electron donor and also oxidizes the phenolic complexes to quinones, which inhibit insect feeding mechanism (Kaur et al., 2017). All living things, especially higher plants, have catalase (CAT). In cells under oxidative stress, it is found in key locations such as mitochondria, peroxisomes, chloroplasts, and cytosol where it aids in catalyzing the breakdown of hydrogen peroxide into water and oxygen (Sharma and Ahmad, 2014). The activity and specific activity level of CAT in leaves of resistant chickpea mutants was 1.79-fold and 1.69-fold, respectively, higher than the susceptible chickpea mutants as reported previously (Kaur et al., 2014). Low CAT activity was found in various diversities, including CM126/15 and CM179/15. PODs, using free radicles, catalyze an oxidation-reduction reaction, which oxidizes or polymerizes many compounds (McGill and Jaeschke, 2013). Lower activity of PODs was observed in CM126/15 and CM137/15. In addition to detoxifying H_2_O_2_, POD also conducts a variety of additional tasks, including the production of free radicals and quinones that are directly poisonous to insects (Zhu-Salzman et al., 2008). It also intercedes the hydroxylcinnamyl alcohol oxidation into free radical intermediates, phenol oxidation, polysaccharide and monomer cross-linking, lignification, as well as suberization, which may lead to the manufacture of antinutritive compounds (He et al., 2011). Hence, the increased POD content in resistant chickpea mutants’ leaves may be improving those chickpea mutants’ physical defenses against insect assault and reducing insect pest damage. Sharma et al. (2016) studied that greater POD activity and total phenol contents may also contribute to resistance to *Helicoverpa armigera* infestation under greater levels of CO_2_. SOD broadly presents metallo enzyme in living organisms. It helps in the disproportionation of superoxide anions to yield hydrogen peroxide and oxygen and neutralizes O_2_ radicals (Yan et al., 2015). A low level of SOD activity was found in CM797/15, and a high level was observed in CM126/15, which made it much more susceptible to biotic stress. SOD plays a significant role in plant stress tolerance mechanism and provides the first line defense against the toxic effects of higher levels of reactive oxygen species (Gharari et al., 2014). It removes O_2_^•−^ by catalyzing its dismutation, one O_2_^•−^ being reduced to hydrogen peroxide and another oxidized to oxygen. It eliminates O_2_^•−^ and declines the risk of OH^•^ production and thus protects the cell from damage (Labudda and Azam, 2014). In living creatures, many hydrolytic enzymes, like esterase, alpha-amylase, and protease, precisely decompose huge molecules into much smaller molecules through process of hydrolysis and add one molecule of water to the substance during this process (Wong et al., 2020). They can also perform as a secondary structure of antioxidants by repairing DNA molecule and by utilizing damaged molecules (Pradedova et al., 2011). Esterase are extensively distributed in various living systems, having significant power to catalyze the hydrolysis and synthesis of ester bonds from different substrates (Zhong et al., 2020).

A low-level esterase activity was noted in CM797/15, and a highest-level esterase activity was found in CM126/15, CM119/15, etc. The current results authenticate that chickpea mutants have greater resistant potential against biotic stress, e.g., pod borer. Normally, TOS of living organisms is used for the estimation of complete oxidation state (Vaiserman et al., 2020). Similarly, the TAS is used to govern the TAC (Sevindik, 2018). A lower level of TOS was noticed in CM17/15, and CM126/15 showed a high value of TOS and TAC. The maximum level of TOS was observed in desi mutant CM3457/91, which was 356 ± 17.5 µM/g s. wt. In studies associated to oxidative stress, MDA content is used as a marker of lipid peroxidation that generally indicates the damage/injuries in membranes of plants (Morales and Munné-Bosch, 2019). A lower level of MDA was observed in CM179/15, and CM126/15 showed intermediate MDA, whereas CM17/15 revealed high MDA. As an alternative of damage, MDA can also help plant to acclimatize by activating some regulatory genes which play role in defense mechanism of plant.

## Conclusion

In conclusion, chickpea mutants CM216-A/15, CM664/15, and CM766/15 depicted highest resilience to CPB due to higher hairiness, better antioxidant defense response and lower levels of hydrolytic enzymes and sugars. TPC was positively associated with pod yield and had negative correlation with pod damage due to CPB. Larvae per plant were positively correlated with TOS. Identified biochemical markers, i.e., TPC, TOS, SOD, and pigments, can be used for screening and selection of CPB-tolerant/resistant mutants. The promising mutants, i.e., CM216-A/15, CM664/15, and CM766/15, can be recommended for general cultivation/use in breeding program as resistance sources for the development of high yielding pod borer–resistant/tolerant chickpea cultivars.

## Data availability statement

The original contributions presented in the study are included in the article/**Supplementary Material**. Further inquiries can be directed to the corresponding author.

## Author contributions

AN: Writing – original draft, Methodology, Formal analysis, Data curation. AH: Writing – review & editing, Supervision, Project administration, Funding acquisition, Conceptualization. TS: Writing – review & editing, Resources, Conceptualization.
